# *Cinnamomum fasciculatum* (Lauraceae), a new species from Guangdong, China

**DOI:** 10.3897/phytokeys.278.199869

**Published:** 2026-08-07

**Authors:** Shuang-Wen Deng, Guan-Yun Huang, Si-Yuan Zeng, Yuan Xu, Rui-Jiang Wang

**Affiliations:** 1 State Key Laboratory of Plant Diversity and Specialty Crops, South China Botanical Garden, Chinese Academy of Sciences, Guangzhou, Guangdong 510650, China Key Laboratory of National Forestry and Grassland Administration on Plant Conservation and Utilization in Southern China, South China Botanical Garden, Chinese Academy of Sciences Guangzhou China; 2 Key Laboratory of National Forestry and Grassland Administration on Plant Conservation and Utilization in Southern China, South China Botanical Garden, Chinese Academy of Sciences, Guangzhou, Guangdong 510650, China State Key Laboratory of Plant Diversity and Specialty Crops, South China Botanical Garden, Chinese Academy of Sciences Guangzhou China; 3 Guangdong Jiangmen Gudoushan Provincial Nature Reserve, Jiangmen 529222, Guangdong, China College of Life Sciences, University of Chinese Academy of Sciences Beijing China; 4 College of Life Sciences, University of Chinese Academy of Sciences, Beijing 100049, China Guangdong Jiangmen Gudoushan Provincial Nature Reserve Jiangmen China

**Keywords:** Chloroplast genome, *
Cinnamomum
*, Lauraceae, morphology, section *Camphora*, taxonomy

## Abstract

*Cinnamomum fasciculatum*, a new species from Guangdong, China, is described and illustrated here. Morphologically, it is most similar to *Cinnamomum parthenoxylon* in having sericeous terminal buds, glabrous branchlets, glabrous leaves, and fruits of similar shape and size. However, *C. fasciculatum* can be readily distinguished by its subterminal inflorescences (vs. axillary and lateral) and a later flowering season (June–July vs. March–May). Phylogenetic analysis based on complete chloroplast genomes also supports that it is a new member of *C.* sect. *Camphora* and is sister to a clade comprising *Cinnamomum mollifolium* and *Cinnamomum tenuipile*. Following the IUCN Red List Categories and Criteria, it is provisionally assessed as Data Deficient (DD) due to insufficient knowledge of its true population status in the wild.

## Introduction

The Lauraceae is one of the representative components of tropical and subtropical evergreen broad-leaved forests, comprising over 3000 species in more than 50 genera (Rohwer 1993; Li et al. 2025). Within Lauraceae, *Cinnamomum* s.l. is one of the largest and most economically important genera, distributed from tropical and subtropical Asia to the western Pacific, with approximately 250 species (IPNI 2026). Morphologically, *Cinnamomum* s.l. is characterized by thyrsoid inflorescences with strictly opposite ultimate cymes, flowers with nine fertile stamens and three staminodes possessing a conspicuous cordate to sagittate glandular head, and a more or less developed cupule (Rohwer 1993; van der Werff 2001; Zeng et al. 2021). Traditionally, *Cinnamomum* s.l. has been divided into sect. *Camphora* Meisn. and sect. *Cinnamomum* (syn. sect. *Malabathrum* Meisn.) (Meisner 1864; Li et al. 1982; Kostermans 1986; van der Werff 2001), comprising ca. 20 and ca. 230 species, respectively. Sect. *Camphora* differs from sect. *Cinnamomum* in its alternate, pinnately to subtriplinerved leaves (vs. (sub)opposite, trinerved leaves), domatia usually present in the axils of lateral veins (vs. domatia absent), and usually perulate buds (vs. non-perulate buds) (Meisner 1864; Li et al. 1982; Zeng et al. 2021).

During field expeditions in 2024 and 2025 in Guangdong, China, we encountered an unknown species of *Cinnamomum* sect. *Camphora*. Subsequent phenological monitoring, morphological and anatomical examination, and phylogenetic analyses confirmed that it represents a new species of *C.* sect. *Camphora*.

## Materials and methods

### Morphological study

Field observations and collections of the putative new species were carried out in 2024 and 2025 in Jiangmen City, Guangdong Province, China. Voucher specimens were deposited at the Herbarium of South China Botanical Garden, Chinese Academy of Sciences (IBSC). We examined physical specimens from IBSC and KUN, as well as digitized type specimens from multiple sources: via the Chinese Virtual Herbarium (CVH, https://www.cvh.ac.cn) from IBSC, KUN, PE, IBK, and LBG; via JSTOR Global Plants (https://plants.jstor.org) from K, E, P, HUH, LINN, BM, NY, and L; and via the TAIF website (https://taif.tfri.gov.tw/tw/index.php) from TAIF. Morphological data were collected from living individuals and specimens. Then, detailed morphological comparisons of the putative new species with the related species were performed based on herbarium specimens, living plants, and relevant taxonomic and floristic literature. Terminology for morphological description followed Beentje (2016).

### Molecular analysis

Three individuals of the putative new species (*Shuang-Wen Deng DSW6891, DSW6894, DSW6898*), collected from the type locality; one sample of *Cinnamomum purpureum* H.G.Ye & F.G.Wang (*Jia-Jia Liu GXLJ0570*) collected from Yangchun, Guangdong; and one sample of *Cinnamomum saxatile* H.W.Li (*Si-Yuan Zeng ZSY694*) collected from Longzhou, Guangxi, were used for molecular sequencing.

Whole genomic DNA was extracted using the modified CTAB method (Doyle and Doyle 1987). Library preparation was performed using the MGIEasy PCR-Free DNA Library Prep Set (MGI-Shenzhen, China), and sequencing was conducted on a DNBSEQ-T7 platform (MGI-Shenzhen, China) with 150 bp paired-end reads.

Chloroplast genome assembly, sequence connection, and annotation were performed using GetOrganelle v1.7.7.1 (Jin et al. 2020), Bandage v0.8.1 (Wick et al. 2015), and Geneious v9.1.4 (Kearse et al. 2012), respectively, with reference to the previously published plastome of *Cinnamomum camphora* (L.) J.Presl (NC_035882). The newly generated plastome sequence was deposited in GenBank.

The phylogenetic dataset comprised 39 chloroplast genome accessions, including five newly generated sequences and 34 accessions downloaded from GenBank (Table 1). Twenty species of sect. *Camphora*, 10 species of sect. *Cinnamomum*, and seven species from closely related genera were sampled according to previous studies (Xiao and Ge 2022; Xu et al. 2025).

**Table 1.** GenBank accession numbers of taxa included in phylogenetic analysis. Sequences generated in this study are marked with asterisks (*).

**Taxa**	**Section within *Cinnamomum***	**GenBank accession number**
*Cinnamomum bodinieri*	sect. *Camphora*	NC_057605
*Cinnamomum camphora*	sect. *Camphora*	MF421523
*Cinnamomum chartophyllum*	sect. *Camphora*	MW421301
*Cinnamomum fasciculatum*	sect. *Camphora*	PZ364433*
*Cinnamomum fasciculatum*	sect. *Camphora*	PZ364434*
*Cinnamomum fasciculatum*	sect. *Camphora*	PZ364435*
*Cinnamomum foveolatum*	sect. *Camphora*	MT621633
*Cinnamomum glanduliferum*	sect. *Camphora*	NC_057217
*Cinnamomum guizhouense*	sect. *Camphora*	NC_085205
*Cinnamomum ilicioides*	sect. *Camphora*	OR264223
*Cinnamomum kanahirae*	sect. *Camphora*	KR014245
*Cinnamomum longepaniculatum*	sect. *Camphora*	MW553040
*Cinnamomum longipetiolatum*	sect. *Camphora*	OR264231
*Cinnamomum micranthum*	sect. *Camphora*	NC_035802
*Cinnamomum migao*	sect. *Camphora*	NC_058709
*Cinnamomum mollifolium*	sect. *Camphora*	MW421302
*Cinnamomum parthenoxylon*	sect. *Camphora*	MH050971
*Cinnamomum purpureum*	sect. *Camphora*	PZ364436*
*Cinnamomum rufotomentosum*	sect. *Camphora*	NC_065107
*Cinnamomum saxatile*	sect. *Camphora*	PZ364437*
*Cinnamomum septentrionale*	sect. *Camphora*	MW421306
*Cinnamomum tenuipile*	sect. *Camphora*	NC_057069
*Cinnamomum appelianum*	sect. *Cinnamomum*	OL943967
*Cinnamomum aromaticum*	sect. *Cinnamomum*	OL943970
*Cinnamomum austrosinense*	sect. *Cinnamomum*	OL943968
*Cinnamomum burmannii*	sect. *Cinnamomum*	OL943969
*Cinnamomum chago*	sect. *Cinnamomum*	MN047449
*Cinnamomum iners*	sect. *Cinnamomum*	OL943974
*Cinnamomum pingbienense*	sect. *Cinnamomum*	OL943977
*Cinnamomum pittosporoides*	sect. *Cinnamomum*	MN047450
*Cinnamomum tamala*	sect. *Cinnamomum*	OL943980
*Cinnamomum verum*	sect. *Cinnamomum*	MT621595
*Aiouea chavarriana*	/	PV553478
*Aiouea montana*	/	OR264207
*Nectandra angustifolia*	/	MF939340
*Ocotea aciphylla*	/	OM135246
*Ocotea guianensis*	/	OM135249
*Sassafras randaiense*	/	MW337246
*Sassafras tzumu*	/	NC_045268

The orientation of the chloroplast genome sequences was manually adjusted, and one inverted repeat region was excluded using Geneious v9.1.4. The sequences were then aligned using MAFFT v7.525 with iterative refinement (--maxiterate 1000) and multi-threading (--thread 256). The resulting alignment was trimmed using TrimAl v1.5 with the automated heuristic model (-automated1) (Capella-Gutiérrez et al. 2009). Finally, a maximum likelihood (ML) tree was constructed using IQ-TREE v3.0.1 with automatic model selection (ModelFinder Plus, MFP) and 1,000 replicates of standard bootstrap analysis.

## Results and discussion

### Morphological comparison

The putative new species, *Cinnamomum fasciculatum* S.W.Deng & R.J.Wang, exhibits the typical morphology of *Cinnamomum* sect. *Camphora*, including alternate, pinnately veined leaves; thyrsoid inflorescences with cymes bearing strictly opposite lateral flowers; bisexual flowers with nine fertile stamens plus three staminodes; cupulate fruits with tepals not persistent; and turbinate pedicels.

Among species of sect. *Camphora*, *C. fasciculatum* is most similar to *Cinnamomum parthenoxylon* (Jack) Meisn. in having sericeous terminal buds, glabrous branchlets, glabrous leaves, and fruits of similar shape and size, but differs in having obovate to obelliptic leaves (vs. elliptic-ovate or narrowly elliptic-ovate), obtuse leaf apex (vs. usually acute or shortly acuminate), subterminal inflorescences arranged in the axils of cataphylls below the terminal vegetative bud or also on axillary brachyblasts (vs. axillary or lateral), and a distinctly later flowering season (June–July vs. March–May) (Table 2).

**Table 2.** Morphological comparison of *Cinnamomum fasciculatum* with *C. micranthum* and *C. parthenoxylon*.

**Character**	***C. fasciculatum***	***C. micranthum***	***C. parthenoxylon***
**Leaf shape**	obovate to obelliptic, rarely elliptic	oblong-elliptic or ovate-elliptic	elliptic-ovate or narrowly elliptic-ovate
**Leaf apex**	obtuse, rounded to subacute	obtuse or apiculate	acute or shortly acuminate
**Lateral veins axils adaxially**	not bullate	bullate	inconspicuously bullate
**Lateral veins axils abaxially**	not dome-shaped	dome-shaped and puberulent	inconspicuously dome-shaped
**Leaf size (cm)**	(4–) 5–8 (–12) × 2.5–3.8(–4.5)	7.5–9.5(–10) × 4–5(–6)	6–12 × 3–6
**Inflorescence**	thyrsoids several, subterminal, arranged in axils of cataphylls below terminal bud, sometimes also on axillary brachyblasts	thyrsoids solitary, terminal, rarely axillary	thyrsoids solitary, axillary or lateral
**Thyrsoid length (cm)**	4.5–6.5	3–5	4.5–8
**Thyrsoid indumentum**	sparsely pilose, especially in lower part, rarely subglabrous	subglabrous or slightly puberulent at base	glabrous
**Fruit shape**	subglobose	ellipsoid	globose
**Fruit size**	8–9 mm long, 8–10 mm in diam.	1.5–2.2 cm long, 1.5–2 cm in diam.	6–8 mm in diam.
**Cupule shape**	obconical	urceolate	obconical
**Cupule apex width (mm)**	ca. 5	9–10	ca. 5
**Flowering season**	June to July	July to August	March to May

*Cinnamomum fasciculatum* also resembles *C. micranthum* in having inflorescences borne at the branch apex, but those of *C. fasciculatum* are arranged below the terminal vegetative bud (vs. terminal inflorescences without a terminal bud in *C. micranthum*). In addition, *C. fasciculatum* bears subglobose fruits (8–9 × 8–10 mm), whereas *C. micranthum* has ellipsoid fruits (1.5–2.2 × 1.5–2 cm). A detailed morphological comparison comprising 12 selected characters is presented in Table 2.

The most distinctive morphological feature of *C. fasciculatum* is its inflorescence position. Within sect. *Camphora*, inflorescences are usually axillary or lateral, rarely terminal. The inflorescence of *C. fasciculatum* is subterminal, i.e., arranged below the terminal buds (Fig. 2B), which is reported here for the first time in sect. *Camphora* (Li et al. 1982, 2008; Ye et al. 2006; Yang et al. 2022b). This inflorescence structure resembles a miniature version of the flowering branches in some sect. *Camphora* species (e.g., *C. parthenoxylon*), but the thyrsoid inflorescences are subtended by caducous bracts rather than leaves.

### Phylogenetic placement and taxonomic implications

**Figure 1.**
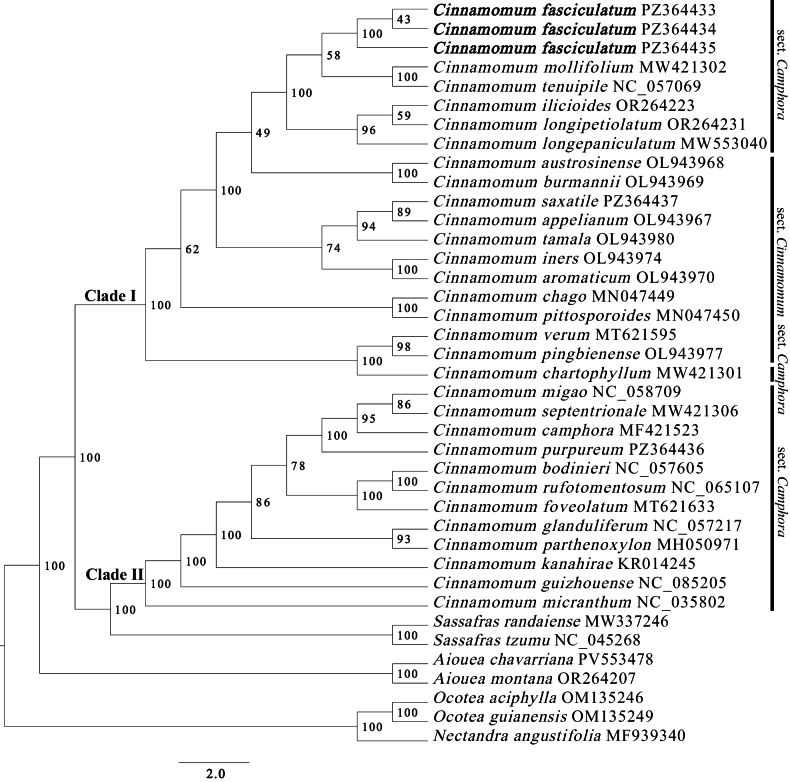
Maximum likelihood phylogenetic tree of *Cinnamomum* based on chloroplast genomes. Bootstrap support values are shown at nodes. The new species is highlighted in bold.

Our maximum likelihood (ML) tree (Fig. 1) inferred from chloroplast genomes resolves *Cinnamomum* into two clades. In clade I, seven species from sect. *Camphora* are nested within sect. *Cinnamomum*; six of them form a monophyletic group, whereas *Cinnamomum chartophyllum* H.W.Li is sister to a subclade including *Cinnamomum pingbienense* H.W.Li and *Cinnamomum verum* J.Presl. In clade II, the remaining 13 species of sect. *Camphora* form a monophyletic group and are sister to *Sassafras*. The three samples of the putative new species, *C. fasciculatum*, cluster together in clade I and further cluster with *Cinnamomum mollifolium* H.W.Li and *Cinnamomum tenuipile* Kosterm.

Yang et al. (2022a) resurrected the genus *Camphora* Fabr. (Fabricius 1759) to accommodate species of sect. *Camphora* s.s., while retaining the generic name *Cinnamomum* for sect. *Cinnamomum* and a few species from sect. *Camphora*. This classification remains controversial, as plastome phylogenetic studies have shown that sect. *Camphora* as traditionally circumscribed is not monophyletic: some species are sister to *Sassafras*, whereas others are nested within sect. *Cinnamomum* (Xiao and Ge 2022; Xu et al. 2025). A phylogenetic analysis based on three nuclear markers (ITS, *LEAFY*, and *RPB2*) by Huang et al. (2016) similarly revealed that three species of sect. *Camphora* (*C. saxatile*, *Cinnamomum longipetiolatum* H.W.Li, and an unidentified species) are nested within sect. *Cinnamomum*. Thus, morphological and molecular delimitations of *Cinnamomum* s.s. and *Camphora* are not fully congruent, and further investigation is needed (Li et al. 2025). Given this uncertainty, and because our phylogenetic result also corroborates this inter-mixed pattern, we adopt a conservative approach and place it in *Cinnamomum* sect. *Camphora*.

## Taxonomic treatment

### Cinnamomum fasciculatum

Taxon classificationPlantaeLauralesLauraceae

S.W.Deng & R.J.Wang sp. nov.

778D12C2-594A-504B-A865-0587D68A68F8

urn:lsid:ipni.org:names:77393773-1

Figs 2, 3, 4

#### Type.

China • Guangdong Province: Taishan City, Mt. Beifengshan, road to Dingzishui Reservoir, 22°16'N, 112°56'E, in evergreen broad-leaved forest, 225 m a.s.l., 9 July 2025 (fl.), *Shuang-Wen Deng*, *Ya-Nan Guo & Peng-Huan Wang 6326* (holotype IBSC1098516; isotype IBSC1098517).

**Figure 2.**
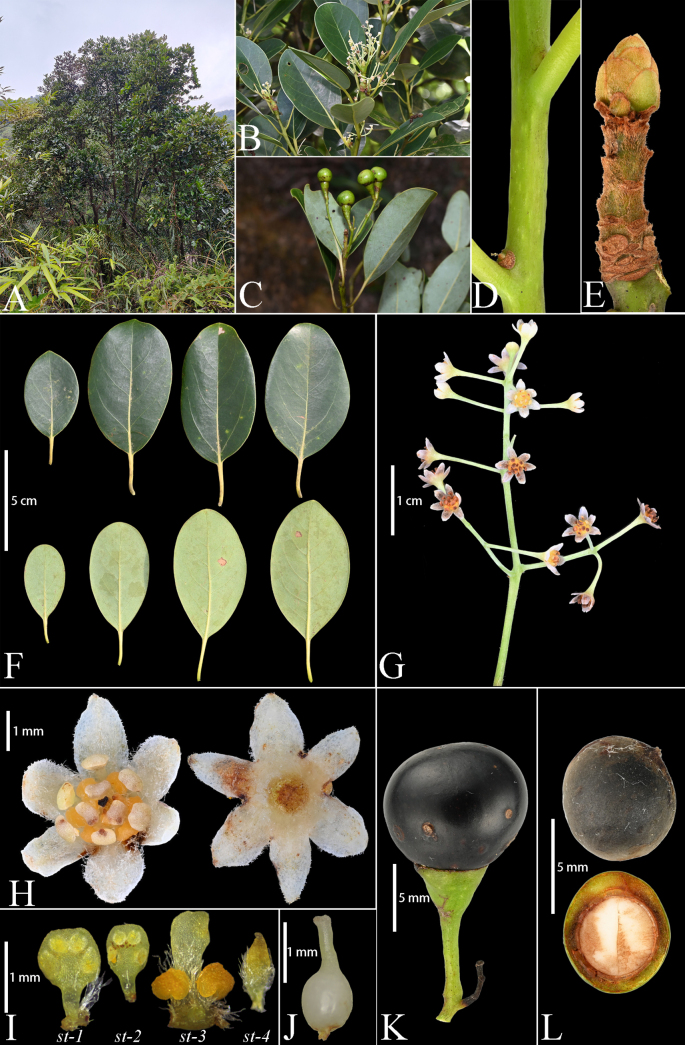
*Cinnamomum fasciculatum* sp. nov. (**B, D–J** from *Shuang-Wen Deng, Ya-Nan Guo & Peng-Huan Wang DSW6326*; **A, C** from *Shuang-Wen Deng & Mei Lu DSW6896*; **K, L** from *Shuang-Wen Deng & Mei Lu DSW6898*) **A**. Habit; **B**. Flowering branch; **C**. Fruiting branches; **D**. Young branchlet; **E**. Terminal bud; **F**. Leaves; **G**. Thyrsoid; **H**. Flower (top view and bottom view); **I**. Stamens; **J**. Pistil; **K**. Mature fruit; **L**. Seed. Photographed by Shuang-Wen Deng.

**Figure 3.**
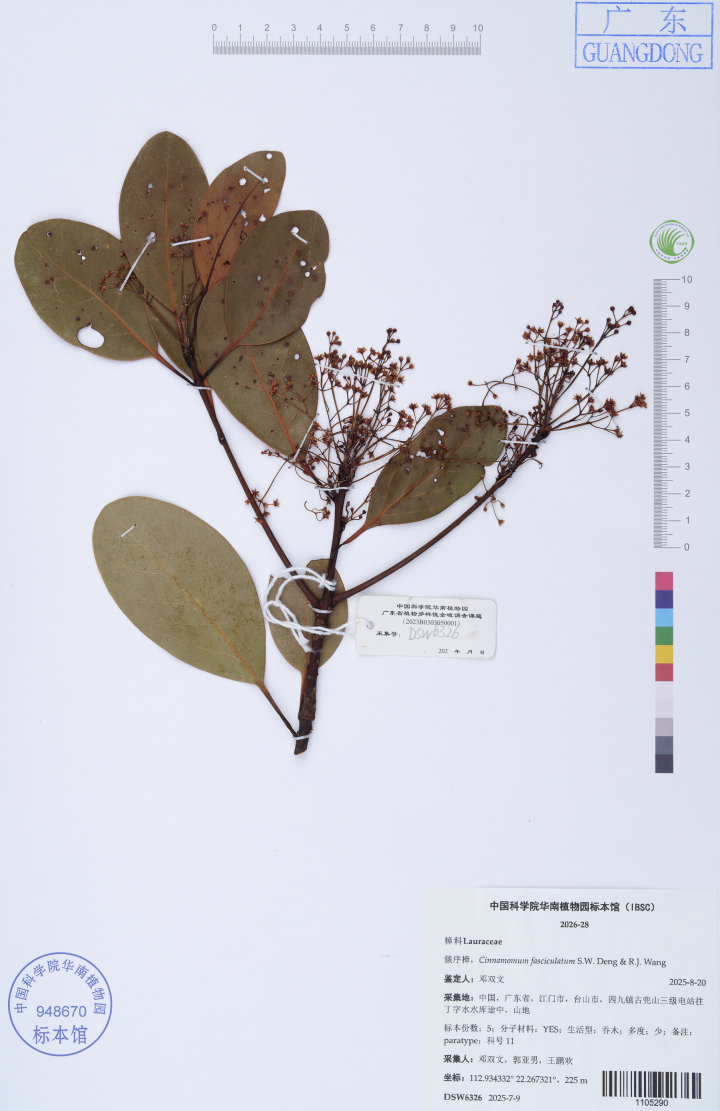
Holotype sheet of *Cinnamomum fasciculatum* sp. nov.

#### Diagnosis.

*Cinnamomum fasciculatum* S.W.Deng & R.J.Wang is morphologically similar to *C. parthenoxylon* (Jack) Meisn. and *C. micranthum* Hayata, but differs by its subterminal inflorescences arranged in the axils of cataphylls below the terminal vegetative bud or also on axillary brachyblasts (vs. axillary or lateral in *C. parthenoxylon*, terminal in *C. micranthum*), obovate to obelliptic leaves with an obtuse apex (vs. elliptic-ovate with acute apex in both), and smaller subglobose fruits (8–9 × 8–10 mm vs. ellipsoid fruits 1.5–2.2 cm long in *C. micranthum*). For a full morphological comparison, see Table 2.

#### Description.

Trees, evergreen, 7–11 m tall, DBH up to 50 cm; crown broadly ovate to globose. ***Trunk*** often low-branching; bark yellow-brown, longitudinally fissured. ***Branchlets*** terete, occasionally slightly angled, puberulent, glabrescent. ***Terminal buds*** ovoid to globose, 4–6 mm long, ca. 4 mm in diam.; bracts semicircular, apex rounded, abaxially densely covered with yellow-brown appressed pubescence, margin ciliate. ***Leaves*** alternate; ***petioles*** glabrous, channeled adaxially, 1.6–2.3 cm long; ***blade*** thinly leathery, obovate to obelliptic, rarely elliptic, (4–) 5–8 (–12) × 2.5–3.8(–4.5) cm, base cuneate, apex obtuse, rounded to subacute, pinnately veined, secondary veins 5–6 pairs, midrib and secondary veins flat adaxially and slightly elevated abaxially, transverse veins and veinlets reticulate, foveolate on both surfaces; adaxially green, abaxially glabrous and glaucous. ***Inflorescence branches*** pubescent. ***Inflorescences*** thyrsoid, several corymbiformly arranged in the axils of cataphylls below the terminal vegetative bud, and sometimes also on axillary brachyblasts, fasciculate-like, 4.5–6.5 cm long, 7–16-flowered, ultimate cymes strictly opposite; bracts caducous; ***peduncles*** sparsely pilose, especially in lower part, rarely subglabrous; ***rachis*** subglabrous; ***pedicels*** slender, 5–6.8 mm long, glabrous. ***Flowers*** bisexual, white-yellow, ca. 5.5 mm in diam.; perianth tube obconical, short, ca. 1.5 mm long; perianth in two whorls, tepals 6, subequal, ovate-elliptic, 1.8–2 mm long, 1 mm wide, glabrous outside and pubescent inside, margin ciliate; ***fertile stamens*** 9, arranged in three whorls, 1^st^ and 2^nd^ whorls ca. 1.1–1.2 mm long, 3^rd^ whorl ca. 1.3 mm long; filaments slightly shorter than or subequal to anthers, 1^st^ and 2^nd^ whorls subglabrous, 3^rd^ whorl villous in the middle and lower part, each stamen of 3^rd^ whorl with two reniform glands at base; anthers yellow, 4-locular, outer two whorls introrse, 3^rd^ whorl extrorse; staminodes 3, sagittate; pistil glabrous, ovary ovoid to ellipsoid, ca. 1 mm long, ca. 0.8 mm in diam., ***style*** glabrous, ca. 0.9 mm long, stigma capitate. Infructescences 2.8–4.5 cm long. ***Fruit*** black when mature, subglobose, ca. 8–9 mm long and 8–10 mm in diam., cupulate, cupules obconical, ca. 5 mm long, apex 5 mm in diam.; pedicels ca. 6.5 mm long; ***seeds*** ellipsoid to subglobose, ca. 11 mm long, 10 mm in diam., yellowish-brown.

#### Phenology.

Flowering from June to July; fruiting from August to November.

#### Etymology.

The specific epithet “*fasciculatum*” refers to the fasciculate-like arrangement of the thyrsoid inflorescences of the new species. The Chinese name of *C. fasciculatum* is proposed as “簇序樟” (Cù Xù Zhāng), a direct translation of the Latin name.

#### Distribution and habitat.

**Figure 4.**
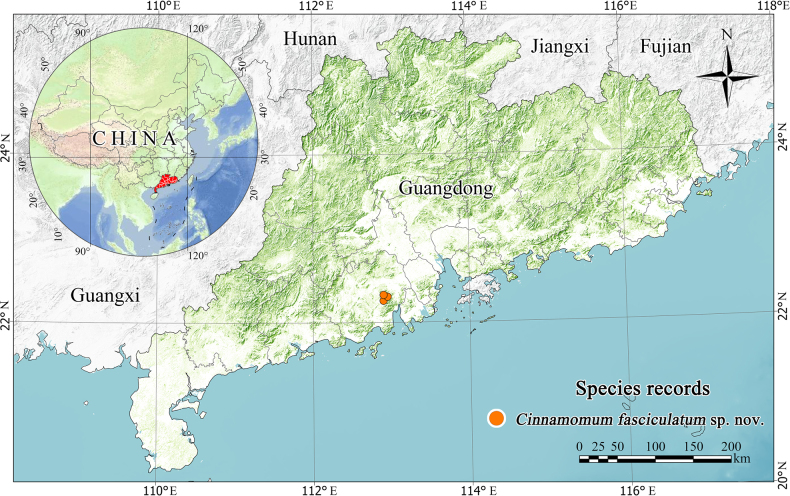
Distribution map of *Cinnamomum fasciculatum*. The national boundary layer was downloaded from the Standard Map Service website (approval number GS(2024)0650; https://cloudcenter.tianditu.gov.cn/administrativeDivision/; accessed July 2026).

*C. fasciculatum* is currently known only from Xinhui District and Taishan County of Jiangmen City, in south-central Guangdong Province, China (Fig. 4). It grows in subtropical evergreen broad-leaved forests at an elevation of 190–300 m a.s.l.

#### Conservation status.

Only two populations of approximately 50 individuals were discovered in Jiangmen City. These two sites are located within the Gudoushan Provincial Nature Reserve. Since little is known about its actual population size and distribution, and further investigation is critically necessary, its conservation status is assessed as “Data Deficient (DD)” at present.

**Additional specimens examined**. China • Guangdong, Xinhui: Gudou Forest Farm, Mt. Wuzhishan, 220 m a.s.l., 28 Sept. 2024 (fr.), *Shuang-Wen Deng, Si-Yuan Zeng & Mei Lu DSW4958* (IBSC); • Taishan: Mt. Gudoushan, Pingnan, 282 m a.s.l., 23 Oct. 2025 (fr.), *Shuang-Wen Deng & Mei Lu DSW6891* (IBSC), *DSW6892* (IBSC); • Mt. Gudoushan, road to Dingzishui Reservoir, 224 m a.s.l. 23 Oct. 2025, *Shuang-Wen Deng & Mei Lu DSW6893*–*DSW6896* (IBSC), *DSW6898* (IBSC); • same locality, 23 Oct. 2025 (fl.), *Shuang-Wen Deng & Mei Lu DSW6897* (IBSC).

## Conclusion

On the basis of morphological and phylogenetic evidence, we confirm that the plant collected from Jiangmen Gudoushan Nature Reserve represents a species new to science and describe it as *Cinnamomum fasciculatum* S.W.Deng & R.J.Wang.

## Supplementary Material

XML Treatment for Cinnamomum fasciculatum

## Additional information

### Artificial Intelligence (AI) use

The authors accept full responsibility for the content of the manuscript, including the disclosure of any use of AI.

No AI tools were used in the preparation of this manuscript.

### Author ORCIDs

S.-W. Deng https://orcid.org/0000-0002-1595-2974

S.-Y. Zeng https://orcid.org/0000-0002-5686-9501

Y. Xu https://orcid.org/0000-0002-7922-6787

R.-J. Wang https://orcid.org/0000-0002-4985-8281

### Data availability

All of the data that support the findings of this study are available in the main text.

## References

[B1] Beentje H (2016) The Kew Plant Glossary: an illustrated dictionary of plant terms, second edition. Royal Botanic Gardens Kew, London, 192 pp.

[B2] Capella-Gutiérrez S, Silla-Martínez JM, Gabaldón T (2009) trimAl: A tool for automated alignment trimming in large-scale phylogenetic analyses. Bioinformatics 25(15): 1972–1973. 10.1093/bioinformatics/btp348 PMC2712344 19505945

[B3] Doyle JJ, Doyle JL (1987) A rapid DNA isolation procedure for small quantities of fresh leaf tissue. Phytochemical Bulletin 19: 11–15.

[B4] Fabricius PC (1759) Enumeratio, Methodica Plantarum. Litteris Ioannis Drimbornii, Helmstadii, 239 pp.

[B5] Huang JF, Li L, van der Werff H, Li HW, Rohwer JG, Crayn DM, Meng HH, van der Merwe M, Conran JG, Li J (2016) Origins and evolution of cinnamon and camphor: A phylogenetic and historical biogeographical analysis of the *Cinnamomum* group (Lauraceae). Molecular Phylogenetics and Evolution 96: 33–44. 10.1016/j.ympev.2015.12.007 26718058

[B6] IPNI (2026) International Plant Names Index. Facilitated by the Royal Botanic Gardens, Kew, Harvard University Herbaria & Libraries and Australian National Herbarium. http://www.ipni.org/ [Retrieved 12 May 2026]

[B7] Jin JJ, Yu WB, Yang JB, Song Y, dePamphilis CW, Yi TS, Li DZ (2020) GetOrganelle: A fast and versatile toolkit for accurate de novo assembly of organelle genomes. Genome Biology 21(1): e241. 10.1186/s13059-020-02154-5 PMC7488116 32912315

[B8] Kearse M, Moir R, Wilson A, Stones-Havas S, Cheung M, Sturrock S, Buxton S, Cooper A, Markovitz S, Duran C, Thierer T, Ashton B, Meintjes P, Drummond A (2012) Geneious Basic: An integrated and extendable desktop software platform for the organization and analysis of sequence data. Bioinformatics 28(12): 1647–1649. 10.1093/bioinformatics/bts199 PMC3371832 22543367

[B9] Kostermans AJGH (1986) A monograph of the genus *Cinnamomum* Schaeff. (Lauraceae) I. Gingkoana 6: 1–171.

[B10] Li HW, Pai PY, Lee SK, Wei FN, Wei YT, Yang YC, Huang PH, Cui HB, Xia ZD, Li JL (1982) Lauraceae. In: Li HW (Ed.) Flora Reipublicae Popularis Sinicae, Vol. 31. Science Press, Beijing, 1–463.

[B11] Li L, Liu B, Song Y, Meng HH, Ci XQ, Conran JG, de Kok RPJ, de Moraes PLR, Ye JW, Tan YH, Liu ZF, van der Merwe M, van der Werff H, Yang Y, Rohwer JG, Li J (2025) Global advances in phylogeny, taxonomy and biogeography of Lauraceae. Plant Diversity 47: 341–364. 10.1016/j.pld.2025.04.001 PMC12146874 40496996

[B12] Li XW, Li J, van der Werff H (2008) *Cinnamomum* Schaeffer. In: Wu ZY, Raven PR, Hong DY (Eds) Flora of China, Vol. 7. Beijing: Science Press, & St. Louis: Missouri Botanical Garden Press, 166–187.

[B13] Meisner CDF (1864) Lauraceae. In: de Candolle A (Ed.) Prodromus Systematis Naturalis Regni Vegetabilis, sive, Enumeratio Contracta Ordinum, Generum, Specierumque Plantarum hucusque Cognitarium, juxta Methodi Naturalis, Normas Digesta, Vol. 15. Paris: Sumptibus Sociorum Treuttel et Würtz, 9–27.

[B14] Rohwer JG (1993) Lauraceae. In: Kubitzki K, Rohwer JG, Bittrich V (Eds) The Families and Genera of Vascular Plants, Vol. 2. Springer-Verlag, Berlin, 366–391. 10.1007/978-3-662-02899-5_46

[B15] van der Werff H (2001) An annotated key to the genera of Lauraceae in the Flora Malesiana region. Blumea 46: 125–140.

[B16] Wick RR, Schultz MB, Zobel J, Holt KE (2015) Bandage: Interactive visualization of de novo genome assemblies. Bioinformatics 31(20): 3350–3352. 10.1093/bioinformatics/btv383 PMC4595904 26099265

[B17] Xiao TW, Ge XJ (2022) Plastome structure, phylogenomics, and divergence times of tribe Cinnamomeae (Lauraceae). BMC Genomics 23: 642. 10.1186/s12864-022-08855-4 PMC9461114 36076185

[B18] Xu J, Zhang H, Yang F, Zhu W, Li Q, Cao Z, Song Y, Xin P (2025) Phylogeny of *Camphora* and *Cinnamomum* (Lauraceae) based on plastome and nuclear ribosomal DNA data. International Journal of Molecular Sciences 26(3): 1370. 10.3390/ijms26031370 PMC11818869 39941137

[B19] Yang Z, Liu B, Yang Y, Ferguson DK (2022a) Phylogeny and taxonomy of *Cinnamomum* (Lauraceae). Ecology and Evolution 12: e9378. 10.1002/ece3.9378 PMC9526118 36203627

[B20] Yang Z, Deng C, Wang L, Ban Q, Yang Y (2022b) A new species of *Cinnamomum* (Lauraceae) from southwestern China. PhytoKeys 202: 35–44. 10.3897/phytokeys.202.76344 PMC9849024 36761812

[B21] Ye YS, Ye HG, Wang FG (2006) *Cinnamomum purpureum* (Lauraceae): A new species from Guangdong, China. Novon: A Journal for Botanical Nomenclature 16(3): 439–442. 10.3417/1055-3177(2006)16[439:CPLANS]2.0.CO;2

[B22] Zeng G, Liu B, Rohwer JG, Ferguson DK, Yang Y (2021) Leaf epidermal micromorphology defining the clades in *Cinnamomum* (Lauraceae). PhytoKeys 182: 125–148. 10.3897/phytokeys.182.67289 PMC8516828 34720625

